# Association between nasal shedding and fever that influenza A (H3N2) induces in dogs

**DOI:** 10.1186/1743-422X-8-1

**Published:** 2011-01-05

**Authors:** Daesub Song, Hyoungjoon Moon, Kwonil Jung, Minjoo Yeom, Hyekwon Kim, Sangyoon Han, Dongjun An, Jinsik Oh, Jongman Kim, Bongkyun Park, Bokyu Kang

**Affiliations:** 1 Viral Infectious Disease Research Center, Korea Research Institute of Bioscience and Biotechnology, Daejon, 305-806, Korea; 2 Research Unit, Green Cross Veterinary Products, Yong-in, 449-903, Korea; 3 Department of Veterinary Medicine Virology Laboratory, College of Veterinary Medicine and BK21 Program for Veterinary Science, Seoul National University, Gwanak-gu, Seoul 151-742, Republic of Korea; 4 National Veterinary Research and Quarantine Service, Anyang, Korea; 5 Bionote, Inc., Hwasung, 443-823, Korea; 6 Research and Development Center, Daewoong Pharmaceutical CO., LTD., 501-2, Samgye-Ri, Pogok-Myun, Kyounggi-Do, 449-814, Korea

## Abstract

**Background:**

Avian origin canine influenza virus was reported in Korea. The dog to dog contact transmission of the avian origin canine influenza virus (CIV) H3N2 and CIV H3N8 was shown by experimental contact transmission. This study was focused on viral excretion and fever in order to elucidate the epidemiological associations which might be helpful to control the disease transmissions in CIV outbreak in dogs.

**Methods:**

An influenza seronegative 10-week-old Beagle dog was experimentally inoculated with the canine influenza virus A/canine/01/2007, subtype H3N2. Eight hours after inoculation, the infected dog was cohoused with seven uninfected Beagle dogs. Clinical signs including fever were recorded for 14 days post inoculation.

**Results:**

The infected dog and four of seven contact dogs in the study showed clinical signs (sneezing, nasal discharge and coughing) during the study. Viral shedding occurred in all of the animals tested and began on 1 to 6 DPI in dogs with clinical signs. Elevated body temperatures above 39.5°C (geometric mean temperature of 39.86°C±0.49) were observed in all symptomatic dogs. The mean viral titer during fever was 2.99 log EID_50_/ml, which was significantly higher than the viral titer detected in the non fever.

**Conclusions:**

The data show that contact dogs with a canine influenza infected dog shed different levels of virus in their nasal excretions and demonstrate that clinical signs, including fever, significantly correlate with the viral shedding.

## Background

Canine influenza virus infection caused severe and acute respiratory symptoms in infected dogs [1-4]. Especially avian origin canine influenza virus was reported in Korea [2]. The experimental or natural infection of type A influenza virus infection of the dogs from human [5,6] and horse [3,7] were reported. The dog to dog contact transmission of the avian origin canine influenza virus (CIV) H3N2 [1] and CIV H3N8 [8] was shown by experimental contact transmission. Regardless of subtype, H3N8 or H3N2 CIV could infect nascent individuals and causes clinical signs [1,8]. The study about experimental transmission of H3N8 CIV in dogs showed that significant relation between the viral load and clinical signs as dictated in textbook [8,9].

Clinical signs caused by influenza virus were diverse respiratory signs including cough, sneezing, pneumonia, myositis, cardiac dysfunctions, and central nervous syndrome[10]. However, fever is the most significant clinical feature in influenza virus infection. It was predicted that the cause of fever would be pyrogenic cytokines including tumor necrosis factor-α, interleukin, and interferon [11].

This study was focused on two points, viral excretion and fever. The relation between clinical signs, especially fever, and viral excretion via nasal shedding in order to elucidate the epidemiological associations, which might be helpful to control the disease transmissions in CIV outbreak in dogs.

## Methods

An influenza-seronegative 10-week-old Beagle dog was experimentally inoculated with the avian origin canine influenza virus A/canine/01/2007, subtype H3N2, which was originally isolated from a pet dog with severe respiratory syndrome [2]. Eight hours after inoculation, the infected dog was cohoused with seven uninfected Beagle dogs in the animal facilities at Green Cross Veterinary Products (Yongin, South Korea). All animal experiments complied with the current laws of South Korea. Animal care and treatment were conducted in accordance with guidelines established by the Green Cross Veterinary Products Institutional Animal Care and Use Committee.

Clinical signs (sneezing, nasal discharge and coughing) and rectal temperatures were recorded daily for 14 days post-inoculation (DPI), and nasal swab samples for the detection of viral shedding were also collected daily over the same time course. Serum samples were collected on days 0, 7, and 14, and antibodies against nucleoprotein (NP) were detected by a commercial canine influenza virus (CIV) competitive ELISA (Animal Genetics, Inc., Suwon, South Korea) [1].

To investigate the correlation between fever and viral shedding, the mean viral titers in the nasal discharge were examined during fever (body temperature >39.5°C) and non-fever stages.

## Results

### Clinical finings

**Figure 1:**
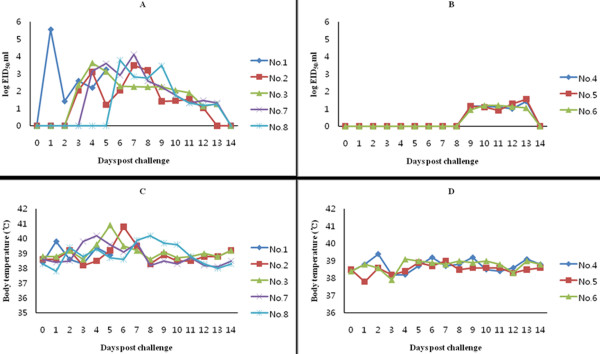
**Levels of CIV shed in the nasal discharge of dogs with and without clinical signs**. The daily levels of viral shedding are expressed as the log EID_50_/ml from five individual dogs with clinical signs (A) and three dogs without clinical signs (B). Body temperatures (°C) were also compared between dogs with (C) and without (D) clinical signs.

The infected dog and four of seven contact dogs in the study showed clinical signs (sneezing, nasal discharge and coughing) during the study, while 3 dogs lacked symptoms. All of the animals seroconverted as assessed by a CIV competitive ELISA (data not shown). Clinical signs were observed from 4 to 8 DPI, and viral shedding was detected earlier in dogs with clinical signs than those without (Figure 1).

### Viral shedding and body temperature

Viral shedding occurred in all of the animals tested and began on 1 to 6 DPI in dogs with clinical signs, but was not detected until day 9 DPI in symptom free dogs. The viral titers shed by dogs with clinical signs were also higher than those shed by asymptomatic dogs. Specifically, viral titers were higher than 3 log EID_50_/ml at any time post-challenge in dogs with clinical signs but remained less than 1.6 log EID_50_/ml in those without clinical signs.

**Figure 2:**
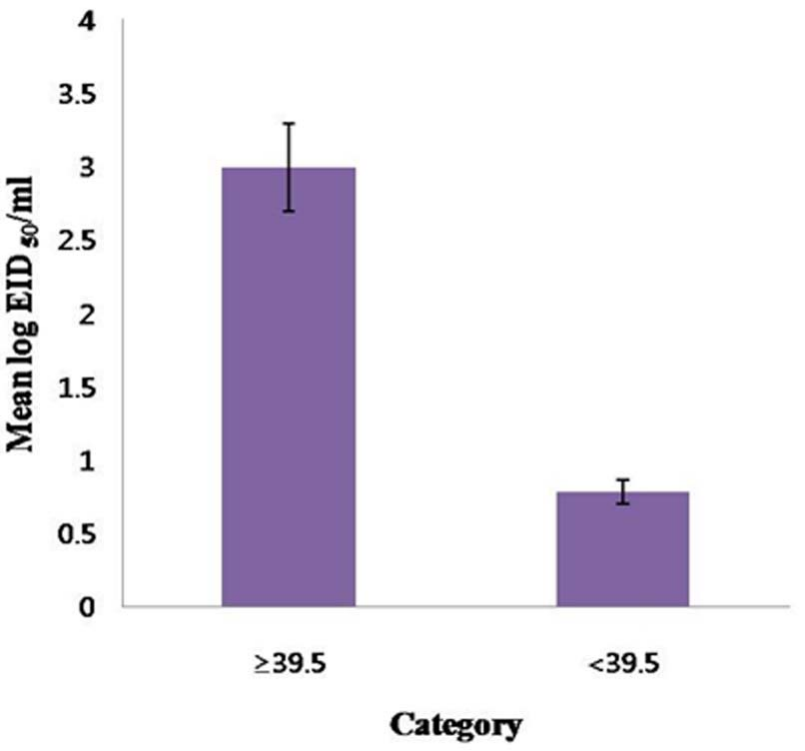
**Comparison of the mean viral titer during fever (>39.5°C) and non-fever stages (<39.5°C)**. The mean viral titer in the presence and absence of fever was 2.99 and 0.78 log EID_50_/ml, respectively, and this difference was statistically significant (p << 0.001, Student's T test.).

Body temperature (°C) was also monitored during the study, and increases in body temperature correlated with the level of viral shedding as well. Elevated body temperatures above 39.5°C (39.86°C ± 0.49) were observed in all symptomatic dogs, while the body temperatures of all dogs without clinical signs remained below 39.5°C (38.68°C ± 0.41). The mean viral titer during fever was 2.99 log EID_50_/ml, which was significantly higher than the viral titer detected in the non fever (0.78 log EID_50_/ml; p << 0.001, Student's T-test) (Figure 2).

## Discussion

Interspecies transmission of influenza viruses, especially highly pathogenic avian H5N1, is a possible threat to global human health [12]. Successful interspecies transmission of the avian influenza virus H3N2 to dogs and subsequent intraspecies transmission between dogs has been reported previously [1,2]. These data suggest that avian influenza viruses can traverse interspecies (avian to mammal) transmission barriers, which in turn suggests the possibility for transmission of influenza viruses from birds to humans.

Since influenza virus was first isolated in 1933 [13], significant attention has been focused on elucidating its structure and genome, the immune responses it elicits, testing protective vaccines, and studying influenza epidemiology. However, information regarding the kinetics of influenza virus during an infection within an individual is limited. Human influenza infections are generally characterized by exponential growth of the virus, which peaks 2 to 3 days DPI, followed by an exponential decrease in the viral load until the virus is undetectable 6 to 8 DPI [14].

During influenza infection, virus shed in nasal and oropharyngeal secretions and dispersed through sneezing and coughing. Since the duration of shedding and the quantity of virus shed are important determinants of infectivity, treatment strategies for influenza should consider not just symptom reduction but also focus on reducing infectivity. The results of this study are also applicable to animal clinics that experience canine influenza cases. Veterinarians should segregate those animals suspected of carrying influenza that show a high fever (>39.5°C) and thus may spread high viral loads by nasal shedding. It has not yet been elucidated why the severity of clinical signs, especially fever, is closely related to levels of viral shedding in infected animals. This should be further studied with regard to the variability of inflammatory and immune responses in CIV-infected hosts. In similar study of viral shedding in human after infection of pandemic H1N1, viral load was maintained at a high level during the febrile period. And pandemic flu-infected patients with pneumonia had a higher viral load than those with mild lesion related with flu. The higher viral shedding load may be a reflection of disease severity, or impaired host defense mechanism, need immediate attention and treatment [15].

The data above show that dogs that commingle with a canine influenza infected dog shed different levels of virus in their nasal excretions. The correlation between the viral shedding and clinical signs of canine influenza H3N2 virus infection would provide important knowledge for epidemiological control and clinical management in terms of infection control strategy.

## Competing interests

The authors declare that they have no competing interests.

## Authors' contributions

DSS: design of the experiments and writing a manuscript, HJM: helped in writing a manuscripts, KJ: participated the correction of manuscripts and support the pathological information, MJY: conducted animal care and sample collection, HKK: conducted data analysis, statistical analysis and drawing the figures, SYH: conducted real time RT-PCR for CIV, DJA: participated writing a manuscript for discussion, JMK: Acquired funding and supplied CIV, BKP: Idea development, BKK: final correction of the manuscript. All authors read and approved the final manuscript.
